# Engineering microbiomes for enhanced bioremediation

**DOI:** 10.1371/journal.pbio.3002951

**Published:** 2024-12-16

**Authors:** Xihui Xu, Jiandong Jiang

**Affiliations:** Department of Microbiology, College of Life Sciences, Nanjing Agricultural University, Key Laboratory of Agricultural and Environmental Microbiology, Ministry of Agriculture and Rural Affairs, Nanjing, China

## Abstract

Bioremediation using synthetic microbiomes offers significant advantages over traditional single‐strain‐based approaches. In this Perspective, the authors outline five important considerations for the rational design of pollutant‐degrading microbiomes for enhanced bioremediation.

## Teaser

Bioremediation using synthetic microbiomes offers significant advantages over traditional single-strain-based approaches. In this Perspective, we outline 5 important considerations for the rational design of pollutant-degrading microbiomes for enhanced bioremediation.

The continuous advance of modern industry and agriculture has resulted in the widespread distribution of organic pollutants (OPs) in our natural environment, raising significant public concern. These pollutants pose serious environmental challenges, threatening both ecosystems and human health.

Microbial bioremediation, which harnesses the metabolic activities of microbes to degrade OPs, is increasingly recognized as a cost-effective and environment-friendly solution for cleaning up contaminated sites, including pesticides (such as glyphosate and atrazine), antibiotics (such as amoxicillin and ciprofloxacin), polycyclic aromatic hydrocarbons (such as naphthalene and phenanthrene), and heavy metals (such as cadmium and lead). Over the past few decades, numerous pure-culture microbial strains capable of degrading OPs have been isolated, providing valuable resources for the remediation of contaminated environments [1]. Common microbial remediation strategies include bioaugmentation (inoculating degrading strains to contaminated sites) and biostimulation (adding nutrients to contaminated sites) [2]. However, the practical application of these strategies often faces challenges. The effectiveness of single-strain-based bioremediation is frequently limited by various factors, including the low survival rate of inoculated exogenous degrading strains due to competition with indigenous community members or nutrient deficiencies, the inability of a single strain to fully catabolize complicated pollutants, the inhibition of metabolic activities by non-target pollutants or their intermediates, and the difficulty in determining necessary exogenous nutrients for effective biostimulation [3–5].

**Fig 1:**
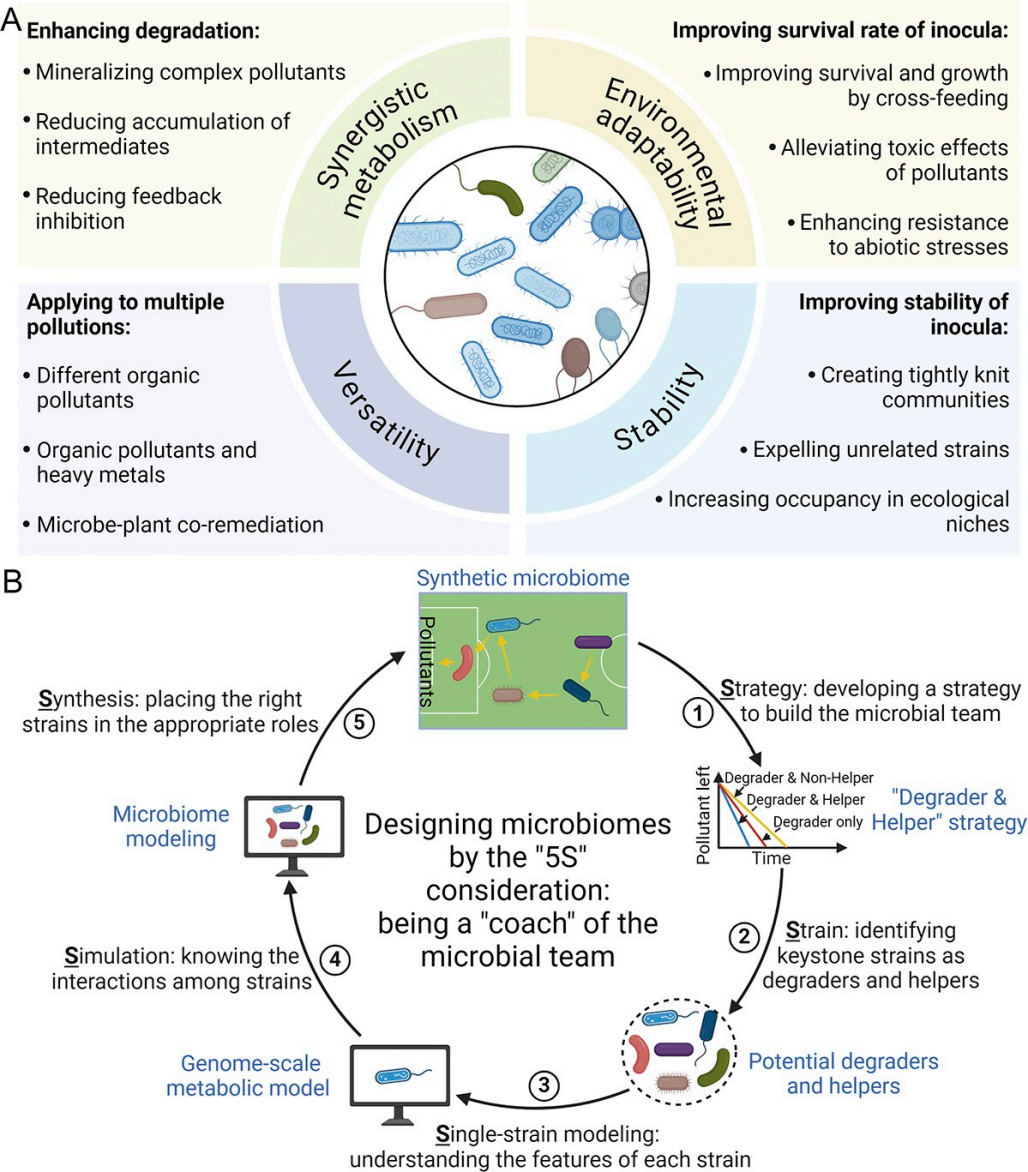
Engineering microbiomes for bioremediation. (A) Potential advantages of microbiome-based bioremediation compared with single-strain-based bioremediation. (B) A schematic depicting the “5S” factors to consider when constructing synthetic microbiome for enhanced bioremediation. Created with BioRender.com.

In contrast, microbiomes—assemblages of diverse microbes—offer significant advantages over traditional single-strain approaches in bioremediation (Fig 1A). For example, microbiomes exhibit extensive genetic and metabolic diversity, enabling the complete degradation of complicated pollutants through synergistic metabolism. This synergy reduces the accumulation of intermediates and minimizes feedback inhibition, which can otherwise hinder degradation activity. Furthermore, cross-feeding within the microbiome promotes the growth of degraders, thereby improving overall degradation efficiency. For example, the atrazine-degrader *Arthrobacter* sp. AT5 secretes aminoethanol, ethylamine, and hypoxanthine during atrazine degradation, which are consumed by the non-degrader *Halobacillus* sp. NY15. In return, strain NY15 secretes ammonium and leucine that are consumed by strain AT5, promoting its growth and atrazine degradation [6]. Inoculating a microbiome consisting of degraders and other functional strains (such as non-degraders functioning as helper strains) into contaminated environments improves the environmental adaptability of the inoculated strains. The helper strains may facilitate biodegradation by providing nutrients through cross-feeding and/or alleviating the toxic effects of pollutants or other abiotic stresses, thereby improving the survival rates and growth of the inoculated degraders. For example, the tetrahydrofuran (THF) degrader *Rhodococcus ruber* YYL is very sensitive to low pH stress, which decreases its growth rate and THF degradation efficiency [4]. However, in a consortium consisting of strain YYL and THF-non-degrader *Bacillus cereus* MLY1, strain YYL produces THF degradation intermediates that are nutrients for strain MLY1, while strain MLY1 consumes acid metabolites produced by YYL elevating pH and helping YYL resist acid stress. MLY1 also consumes THF degradation intermediates, alleviating the product limitation of YYL, and produces essential micronutrients that promote the growth of YYL [4]. In addition to interactions between microbiomes and their external environment, interactions within microbiomes (whether direct or indirect) are also beneficial in promoting the establishment of stable community structures. For example, cross-feeding between community members can create tightly knit communities that outcompete unrelated strains, thus enhancing the competitiveness of the inoculated microbiome against indigenous microbes and increasing their occupancy in specific ecological niches. Lastly, microbiomes are capable of executing more diverse functions than single strain of microbes. By incorporating strains that target different OPs, a microbiome can be engineered to effectively mineralize mixtures of various OPs. Additionally, designing microbiomes with both OP-degrading and heavy metal (HM)-tolerant strains can address environments contaminated with both OPs and HMs.

Importantly, microbiomes are also compatible with microbe-plant co-remediation strategies when applied to the rhizosphere, the narrow region of soil that surrounds and is influenced by plant roots. Engineered rhizosphere microbiomes can perform multiple functions—such as OP degradation, HM immobilization, phytopathogen inhibition, plant growth-promotion, and stress tolerance enhancement—thereby enhancing the efficiency of pollutant degradation while reducing the reliance on chemical pesticides and fertilizers [7].

Despite the promising prospects of microbiome-based bioremediation, a critical question remains: Can we directly use natural microbiomes for this purpose? Unfortunately, the answer is no. Increasing evidence suggests that the community structure of microbiomes not only influences the efficiency of pollutant degradation but also determines the ultimate degradation products [8]. Naturally occurring microbial assemblages are not necessarily optimized for OP degradation, and the optimal community structure can vary with environmental conditions. Therefore, it is essential to design and optimize the community composition and structure of microbiomes to develop effective bioremediation strategies.

Achieving this goal necessitates the development of new technologies that link microbiome structure and metabolic interactions within the microbiome to physiological functions.

However, understanding the metabolic interactions, even within simple microbiomes (such as two-member combinations), and correlating them with physiological performances, remains challenging. This complexity is further compounded in natural microbiomes existing within dynamic environmental contexts.

Synthetic biology, which involves engineering existing organisms or creating new life forms, is recognized as a revolutionary technology capable of addressing significant human challenges [9]. Extending the principles of synthetic biology from single-cell strategies to multicellular systems, synthetic microbiomes enable the de novo design and manipulation of microbiomes [10]. This approach aims to distribute different metabolic pathways across multiple strains, constructing optimal combinations for specific purposes. Thus, synthetic microbiomes represent an attractive avenue for the rational design of microbiomes for enhanced bioremediation.

Yet, most researchers still resort to trial-and-error approaches to determine the optimum design of synthetic microbiomes. Here, we outline 5 important considerations when designing and constructing enhanced pollutant-degrading microbiomes (Fig 1B). In this process, we function as “coaches” for the synthetic microbial “team.” As coaches, our responsibilities include developing strategies, identifying suitable team members, understanding the characteristics of each member, analyzing their interactions, and placing the right members in the appropriate roles.

**Strategy**: Our experiments, along with many other studies, have demonstrated that combining degrading strains with specific helper strains can significantly enhance pollutant-degradation, an improvement not observed with non-helper strains [6,11]. Therefore, the “degrader & helper” strategy could be employed to construct efficient pollutant-degrading microbiomes.

**Strain**: For most common OPs, degrading strains are usually available [1]. The practical task for a “coach” is to identify and isolate indigenous strains from the polluted sites as potential helpers. The use of indigenous strains aims to enhance the adaptability of the synthetic microbiome to the polluted environments. Stable isotope probing (SIP) technology can identify in situ helpers by feeding a consortium containing degraders and indigenous strains with ^13^C-labeled OPs, thus identifying potential helpers involved in degradation [12]. Given the challenges and costs associated with isotopically labeled OPs, alternative methods should be explored. For example, treating in situ consortium with degraders and OPs and then analyzing the dynamic changes in the consortium can help to identify indigenous helper strains that respond to the treatment, as indicated by variations in their abundance [5].

**Single-strain modeling**: Genome-scale metabolic models (GSMMs), which mathematically represent a cell’s entire metabolic network, can elucidate the characteristics of each strain.

These models provide information on metabolic network topology, enzymatic reactions and their directions, substrate-product stoichiometry, and more [13].

**Simulation**: Simulating the metabolic and growth performance of different strain combinations can predict the community structures of the optimal combinations. Tools like SuperCC (https://github.com/ruanzhepu/superCC) can simulate interactions between degraders and helpers, effectively identifying optimal combinations for efficient pollutant degradation [9]. These simulations can also predict nutrient additives that could enhance degradation, providing a basis for biostimulation.

**Synthesis**: Constructing synthetic microbiomes with the predicted functional strains allows for their further testing and application [5].

While the “5S” factors hold promise for bioremediation, several challenges remain.

The applicability of the “degrader & helper” strategy to all OPs needs further investigation. In addition, isolating functional strains identified by culture-independent high-throughput sequencing technology can be challenging, necessitating advances in strain isolation techniques. Alternatively, constructing synthetic microbiomes using available strains could serve as a feasible compromise. Designing synthetic microbiomes also needs high-quality GSMMs of numerous strains, and the accuracy of predictions heavily depends on model quality. Developing automated methods for constructing and curating models and building comprehensive databases of GSMMs are crucial. Another issue is that metabolic interactions between community members are complex, easily influenced by environmental factors, and may change over time. Integrating multi-omics data (such as transcriptomics, proteomics, and metabolomics) and new analytical technologies (such as machine learning) into modeling could increase predictive accuracy. Lastly, it will be necessary to consider strategies for maintaining microbiome stability, which could enhance and prolong the efficiency of the microbial community. To this end, the use of indigenous strains, particularly those from contaminated soils, is recommended due to their ability to rapidly adapt to environmental changes and maintain community stability. Strains that have complementary, rather than competing, metabolic pathways could be selected to reduce community fluctuations due to resource competition. It is advisable to choose strains with stable genetic traits to avoid genetic drift, ensuring long-term stability. It is also beneficial to introduce multiple strains that perform the same function, as functional redundancy will contribute to community stability, providing backup capabilities in case of strain failure.

We envision that the use of synthetic microbiomes will help to overcome existing bottlenecks in designing enhanced pollutant-degrading microbiomes, fully realizing the potential of microbial bioremediation.
